# Reinterpreting olive bud dormancy

**DOI:** 10.1093/jxb/erae353

**Published:** 2024-08-22

**Authors:** Mercedes Arias-Sibillotte, Michael J Considine, Santiago Signorelli

**Affiliations:** Department of Plant Production, School of Agriculture, Universidad de la República, Montevideo, 12900, Uruguay; School of Molecular Sciences, The University of Western Australia, Crawley, WA 6009, Australia; Department of Primary Industries and Regional Development, Perth, WA 6000, Australia; School of Molecular Sciences, The University of Western Australia, Crawley, WA 6009, Australia; Food and Plant Biology group, School of Agriculture, Universidad de la República, Montevideo, 12900, Uruguay; Heinrich Heine University Düsseldorf, Germany

**Keywords:** Bud, dormancy, olive, flowering, quiescence

**Current evidence suggests that olive buds do not undergo physiological dormancy; however, the parallel processes of floral induction and bud quiescence may share conserved molecular pathways, which possibly confounds interpretation of existing data.**

Olive (*Olea europaea* L.) is a millennial crop that originated in the Mediterranean region, and its phenology is adapted to seasonal cues of temperature and photoperiod (Loumou and Giourga, 2003; Ferrara and Ingemark, 2023). The benefits of olive oil for human health have promoted its consumption, and for this reason, olive production has been extended to climatic conditions very different from those found in the Mediterranean region, creating challenges for the effective management of production (Torres *et al.*, 2017; Conde-Innamorato *et al.*, 2019; Sánchez-Martínez and Garrido-Almonacid, 2019). In addition, progressive climate change and increases in the frequency of extreme events exposes the crop to great challenges (Ben-Ari *et al.*, 2021).

Olive trees display episodic growth, typically two flushes of extension growth, occurring in late spring or summer and late autumn or winter, separated by quiescence of the terminal bud (Sanz‐Cortés *et al.*, 2005; Aïachi Mezghani *et al.*, 2008). A cold requirement for good flowering was established in the mid-20th century (Hackett and Hartmann, 1963, 1967; Hartmann and Whisler, 1975). This evidence led to studies on the effect of low temperatures on bud sprouting or dormancy release (Rallo and Martin, 1991; Ramos *et al.*, 2018; Rubio-Valdés *et al.*, 2022; Inês *et al.*, 2023) and the modelling of cold requirements to predict olive grove phenology (De Melo-Abreu *et al.*, 2004; López-Bernal *et al.*, 2020).

The nature of the quiescence, its environmental plasticity, and its importance for flowering and production have resulted in considerable attention to the study of bud dormancy. Different methodological strategies have led to descriptions of the environmental requirements for dormancy onset and release in deciduous trees (Fadón *et al.*, 2020; Singh *et al.*, 2017).

In olive trees, which are evergreen, the first sign of microscopic differentiation of the reproductive meristem occurs after winter (Fabbri and Benelli, 2000; Kitsaki *et al.*, 2010; Haberman *et al.*, 2017; Ramos *et al.*, 2018). Thus, reproductive and vegetative buds cannot be microscopically distinguished during winter, unlike in many economically important deciduous trees. For this reason, most studies during the winter rest phase simultaneously analyse floral induction, release from dormancy or quiescence, and the initiation of differentiation. This feature of the annual cycle of olive constitutes a great challenge for experimental design, and it is unlikely to be overcome until more comprehensive genetic approaches are applied, in order to differentiate the states and fate of the buds during winter.

The olive tree displays a biennial or alternate bearing behaviour (the tendency to bear fruit in two-year cycles), which poses logistical challenges for commercial production (Monselise and Goldschmidt, 1982; Goldschmidt, 2013; Smith and Samach, 2013; Goldschmidt and Sadka, 2021). Hence, understanding the factors controlling bud fate is essential to address environmental challenges through good agricultural practices and genetic programs. Moreover, these concepts appear to be confounded in literature of tropical or subtropical fruit trees, such as mango and avocado, where floral induction displays facultative requirements for cold.

In this Viewpoint, we re-evaluate the interpretation of data presented in the literature of olive bud dormancy. To do this, we first revisit the basic concepts of dormancy to avoid the confusion between reproductive bud dormancy and floral induction present in the literature, and then focus on bud burst, regardless of the reproductive or vegetative nature, to draw conclusions about olive bud dormancy.

## Basic concepts to uniform language and interpretation

Dormancy (syn. endo-dormancy) is an entrained physiological mechanism that allows perennial buds to survive adverse environmental conditions by calibrating annual growth with predictable seasonal change in temperatures and/or photoperiod. It is typically defined as the failure to resume growth even when conducive conditions of light and temperature are provided (Singh *et al.*, 2017); however, the requirement for entrainment is often facultative, as in grapevine (Considine and Considine, 2016). As dormancy is defined by the absence of change, the need to distinguish it from other conditions that repress growth, including suppression (para-dormancy) or limiting climate conditions (eco-dormancy) is often overlooked. The latter two conditions can be viewed as opportunistic, in that once the limiting factor is removed, growth will resume without delay. Experimentally, this is often achieved by taking single or multiple node cuttings containing the buds and placing them in warm conditions. If buds burst, they are considered not to be dormant, whereas if they do not burst, they are considered dormant. However, the duration of the experiment is important, recalling that in many species the expression of dormancy, or the need for chilling to overcome dormancy, is facultative. Thus, mortality or ‘time to event’ approaches, such as the time to 50% bud burst, can be more informative. Such an approach can provide additional clarity, particularly as the depth of dormancy should be distinguished from the bud burst rate. Finally, the use of a positive control, such as the dormancy-breaking chemical hydrogen cyanamide, assists in distinguishing dormancy from viability.

## Do olive tree buds enter dormancy?

Dormancy studies of olive trees have been carried out to date mainly quantifying the percentage of budburst when explants or whole plants are placed in forcing conditions (Rallo and Martin, 1991; Ramos *et al.*, 2018; López-Bernal *et al.*, 2020; Rubio-Valdés *et al.*, 2022). There are two fundamental elements that lead us to question whether there is a true dormancy in the olive trees. One is the high percentage of bud burst immediately upon transfer to forcing conditions. Ramos *et al.* (2018) showed that defoliation and forcing conditions induced a 100% vegetative bud burst. Defoliation is used to remove the repressive effect of other organs, whereas forcing conditions are used to remove the environmental conditions arresting growth. In this sense, what is known as para-dormancy or eco-dormancy is removed. Since the buds were able to burst, we conclude that the buds do not undergo true dormancy. The second element that we want to highlight is that bud exposure to forcing conditions prior to or early in winter resulted in bud burst only as vegetative buds and not as reproductive buds. This could be due to the lack of FLOWERING LOCUS T (FT) protein in the buds, by either the absence of leaf produced-FT or the lack of cold to induce *OeFT2* locally in the bud (Haberman *et al.* 2017). When defoliation and forcing conditions were applied to explants later, in the late winter, the number of buds resulting in reproductive organs was much greater (Ramos *et al.*, 2018; Rubio-Valdés *et al.*, 2022). The only element where some type of dormancy can be shown is the difference in the sprouting rates of vegetative buds between the buds removed from the field at the beginning or at the end of winter. Vegetative buds from foliated shoots of 1-year-old potted plants take longer to sprout when removed early in the winter (López-Bernal *et al.*, 2017, 2020). The same authors point out the possibility that the reserves accumulated in the buds during the winter period could determine higher development rates in the late winter bud sprouting (López-Bernal *et al.*, 2020).

**Fig. 1.**
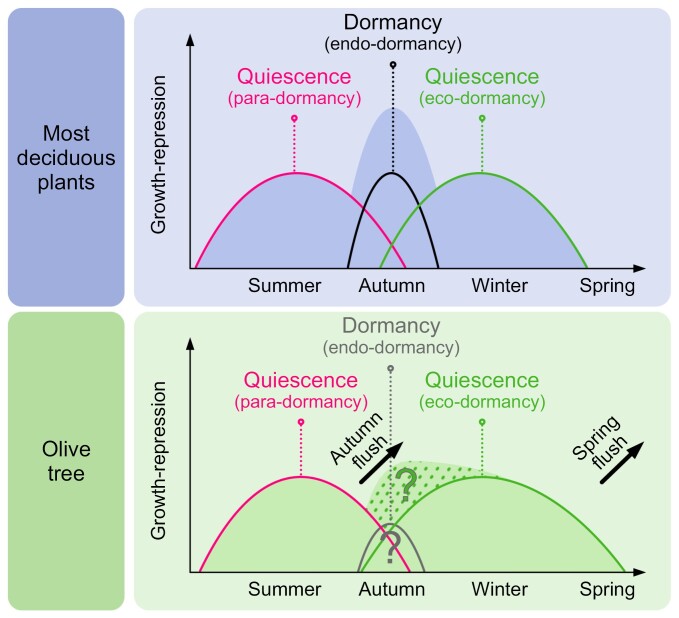
Comparison between the general model for dormancy and quiescence status of buds for most deciduous plants and the olive tree. Deciduous plants originating from northern regions, such as apple and grapevines, have a clear true dormancy with different levels of depth that will not allow them to burst even under optimal growth conditions. However, the evidence suggests that buds of the olive tree, which is originally from the Mediterranean region where prolonged cold periods are rare, do not have a physiological dormancy and their growth is limited either due to the repressor effect of leaves and fruits or due to the lack of optimal conditions to resume growth. Thus, two flushes of growth, in late summer–autumn and spring, occur in olive trees.

The evidence analysed helped us to understand that olive buds undergo a process of para-dormancy (as defoliation allows burst) and then a period of eco-dormancy. This ensures that even in the absence of true dormancy, olive buds will not burst in winter conditions (Fig. 1). Likewise, as olive is a non-deciduous species, the quiescent state of its buds prior to winter will be prolonged as there will be always leaves and/or fruits exerting a repressor effect on axillary buds (Fig. 1). By late winter, this period of quiescence is completed and the microscopic differentiation to vegetative structures (in apical and lateral buds) or to inflorescence (in lateral buds only) begins (Castillo-Llanque and Rapoport, 2011). This phenological stage is identified as a swollen bud, which corresponds to stage 51 of the BBCH scale (Sanz‐Cortés *et al.*, 2005) and occurs from February in the northern hemisphere. Then the buds sprout and macroscopic differentiation continues until anthesis and full bloom, stage 65 (Fabbri and Alerci, 1999; De la Rosa *et al.*, 2000; Kitsaki *et al.*, 2010; Haberman *et al.*, 2017; Ramos *et al.*, 2018).

## Cold and flowering in olive: a seasonal growth view

Unlike several deciduous trees, for olive there are limited molecular data to support the use of markers for dormancy, such as the expression of *DORMANCY ASSOCIATED MADS Box* (*DAM*) genes or *FT* genes responding to changes in temperatures and photoperiod (Singh *et al.*, 2017; Lloret *et al.*, 2018; Wang and Ding, 2023). In evergreen trees, much of our knowledge at the molecular level is limited to the process of flowering rather than dormancy, and how cold induces the expression of *FT* genes to promote flower induction (Rehman *et al.*, 2023). In olive, Haberman *et al.* (2017) demonstrated that cold promotes flowering via *OeFT* because (i) cold induces *OeFT* expression; (ii) treating olive buds with warm temperatures in winter completely suppressed flowering; and (iii) transgenic plants overexpressing *OeFT* flowered out of season, meaning that cold is not needed *per se* to induce flowering, but rather to induce *FT* expression. It is worth noting that the increase in the expression of *OeFT* genes caused by cold is much more pronounced when occurring in OFF trees (trees without fruit) than in ON trees (trees bearing fruit), where the increase is more subtle (Haberman *et al.* 2017). Moreover, the induction of *OeFT* expression and flowering by cold was shown to be independent of the season; however, this effect was less pronounced when cold was applied in summer, suggesting that factors other than *FT* expression were required to induce good flowering (Haberman *et al.* 2017). The dependency of *FT* expression on cold was further supported in different varieties of olive trees (Engelen *et al.* 2023). In other evergreen species such as litchi (Lu *et al.*, 2022), avocado (Ziv *et al.*, 2014; Acosta-Rangel *et al.*, 2021; Ahsan *et al.*, 2023), mango (Gafni *et al.*, 2022), and citrus (Agustí *et al.*, 2022), flowering is also induced by low temperatures involving paralogues of the *FT* gene. In fact, in perennials five different signalling pathways are considered to modulate flowering, including vernalization, photoperiod, gibberellin, aging, and an autonomous pathway. These signalling pathways involve multiple genes, some of which are specific while others are shared (for a detailed review see Rehman *et al.* 2023; Wang and Ding, 2023). The photoperiod pathway involves FT and CONSTANS (CO) as critical proteins regulating flowering (An *et al.*, 2004; Valverde *et al.*, 2004; Hayama *et al.*, 2017), and evidence suggests these transcription factors also play key roles in growth arrest and resumption of growth in perennials (Almada *et al.* 2009; Rinne *et al.*, 2011). Most notably in poplar species there is extensive evidence that *FT* paralogues regulate the annual cycle of woody species (Lloret *et al.*, 2018; André *et al.*, 2022; Li *et al*, 2022; Nilsson, 2022; Rehman *et al.*, 2023; Wang and Ding, 2023), suggesting that FT is an ‘integrating protein’ for environmental factors, temperature, and photoperiod. Interestingly, gene duplication has resulted in functional divergence of poplar *FT1* and *FT2* paralogues, which are transcriptionally regulated by different environmental stimuli and function in regulating different growth processes (André *et al.*, 2022). The absence of similar genetic data in olive species has restricted the ability of the scientific community to dissect the physiological processes governing flowering and bud quiescence. The advent of more efficient gene editing approaches, together with careful experimental design, including positive controls for growth forcing studies, will advance our understanding of the reproductive biology of olive and also assist refinement of tools to improve olive production.
